# Effect of a structured psycho-oncological screening and treatment model on mental health in cancer patients (STEPPED CARE): study protocol for a cluster randomized controlled trial

**DOI:** 10.1186/1745-6215-15-482

**Published:** 2014-12-10

**Authors:** Susanne Singer, Helge Danker, Susanne Briest, Arne Dietrich, Andreas Dietz, Jens Einenkel, Kirsten Papsdorf, Florian Lordick, Jürgen Meixensberger, Joachim Mössner, Dietger Niederwieser, Torsten Prietzel, Franziska Schiefke, Jens-Uwe Stolzenburg, Hubert Wirtz, Anette Kersting

**Affiliations:** Institute of Medical Biostatistics, Epidemiology, and Informatics (IMBEI), Division of Epidemiology and Health Services Research, University Medical Center Mainz, Obere Zahlbacher Straße 69, 55131 Mainz, Germany; Department of Psychosomatic Medicine and Psychotherapy, University Medical Center, Semmelweisstraße 10, 04103 Leipzig, Germany; Breast Cancer Center, University Medical Center, Liebigstraße 18, 04107 Leipzig, Germany; Department of General Surgery, University Medical Center, Liebigstraße 18, 04107 Leipzig, Germany; Department of Otolaryngology, University Medical Center, Liebigstraße 18, 04107 Leipzig, Germany; Department of Obstetrics and Gynecology, University Medical Center, Liebigstraße 18, 04107 Leipzig, Germany; Department of Radiation-Oncology, University Medical Center, Liebigstraße 18, 04107 Leipzig, Germany; University Cancer Center, University Hospital Leipzig, Liebigstraße 20, 04107 Leipzig, Germany; Department of Neurosurgery, University Medical Center, Liebigstraße 18, 04107 Leipzig, Germany; Department of Gastroenterology, University Medical Center, Liebigstraße 18, 04107 Leipzig, Germany; Department of Hematology and Oncology, University Medical Center, Liebigstraße 18, 04107 Leipzig, Germany; Department of Orthopedics, University Medical Center, Liebigstraße 18, 04107 Leipzig, Germany; Department of Maxillofacial Surgery, University Medical Center, Liebigstraße 18, 04107 Leipzig, Germany; Department of Urology, University Medical Center, Liebigstraße 18, 04107 Leipzig, Germany; Department of Pneumology, University Medical Center, Liebigstraße 18, 04107 Leipzig, Germany

**Keywords:** Oncology, Randomized controlled trial, RCT, Cluster randomized trial, Psycho-oncology, Mental health, Screening, Healthcare

## Abstract

**Background:**

High levels of emotional distress in cancer patients often goes unnoticed in daily clinical routine, resulting in severe undertreatment of mental health problems in this patient group. Screening tools can be used to increase case identification, however, screening alone does not necessarily translate into better mental health for the patient. Doctors play a key role in providing basic emotional support and transferring the patients in need of such specific support to mental health professionals. This study investigates whether a stepped care model, combining screening, doctor consultation and professional psycho-oncological service in a structured way, improves the emotional wellbeing of cancer patients.

**Methods/Design:**

This study is a cluster randomized trial with two parallel groups (intervention vs. care as usual), set in an academic hospital. Participants are cancer patients, a total of 1,000 at baseline. The intervention consists of stepped psychosocial care. Step one: screening for distress, step two: feedback of screening results to the doctor in charge of the patient and consultation with the patient, and step three: based on a shared patient-doctor decision, either transferal to the consultation liaison (CL) service or not. The outcome will be emotional well-being half a year after baseline, ascertained with the Hospital Anxiety and Depression Scale. Randomization will be done by the cluster randomization of wards.

**Discussion:**

Mental health problems not only cause emotional suffering but also direct and indirect costs. This calls for timely and adequate psychosocial support, especially as we know that such support is effective. However, not every cancer patient can and must be treated by a mental health professional. Allocating limited resources most sensibly and economically is of crucial importance for our healthcare system to ensure the best quality of care to as many patients as possible. It is the hope of the STEPPED CARE trial that this model is both effective and efficient, and that it can be implemented in other hospitals as well, if proven to be effective.

**Trial registration:**

Clinical Trials Register (Clinicaltrials.gov) identifier: NCT01859429 registration date 17 May 2013.

## Background

Psychosocial distress in cancer patients causes significant financial and emotional costs. It is associated with high utilization of health services, low quality of life and increased mortality [1, 2]. Meta-analyses show that one third of all cancer patients in acute clinics suffer from severe emotional distress and psychiatric comorbidity [3–5]. Unfortunately, such distress often goes undetected by healthcare providers, doctors and nurses alike [6–8]. This can result in considerable undertreatment of mental health problems [9]. Verdonck-de Leeuw *et al*. found in their prospective study that only 21% of highly distressed cancer patients were referred to a psycho-oncologist or a community worker [10]. We saw in one of our studies that only 9% of those cancer patients with a comorbid mental health condition were referred to a mental health specialist within three months of the diagnosis [11].

Such poor uptake of mental healthcare can only partly be explained by patients’ denial of emotional problems or hesitation to seek professional help. On the contrary, if patients are requested to indicate with whom they would like to speak about their emotional problems, one third of patients express the wish to consult a psycho-oncologist, and more than 80% request their physician [12].

It has been shown that if patients are asked to complete a short questionnaire to report psychosocial problems, under-diagnosis of poor mental health can be considerably decreased [7, 13, 14]. However, better identification of distressed patients does not necessarily translate into better patient care and, eventually, to improved mental health. For example, implementing screening for depression into the clinical routine did not improve mental health nor did it reduce supportive care needs in a clinical trial [15], at least not for the entire group of patients; only those with medium or high levels of distress benefited from the screening. Velikova *et al*. found in their three-arm trial (arm one: screening and feedback to the physician; arm two: screening only, no feedback and arm three: no screening) that completing a questionnaire was the crucial element for improving quality of life in cancer patients, whereas the feedback to the physician improved mental health [16].

It thus seems that patients’ mental health can be improved by a smart combination of routine screening and the involvement of healthcare professionals. It is the aim of the current study to test the effect of such a healthcare model.

### Aim and objectives

This study examines the effect of stepped psychosocial care (screening, consultation with a doctor and referral to psychosocial services) on mental health in cancer patients who are admitted for oncological treatment in a hospital.

The objectives are:

1. to determine the feasibility of integrating a new strategy to detect and treat mental health problems of cancer patients in different clinics and wards (stepped care);
2. to investigate the effect of this new strategy (stepped care) on patients’ mental health;
3. to examine the medium-term (six-month) effects of the new strategy (stepped care).

The primary endpoint is patient-reported mental health. Secondary endpoints are social functioning, psychiatric comorbidity, satisfaction with care and utilization of healthcare.

## Methods/Design

### Design

Experience from previous studies teaches us that investigating complex behavioral interventions with ‘simple’ randomized trials can create misleading results. If, for example, students in a class are individually randomized to either be trained to stop smoking or not, the outcome ‘change in frequency of smoking’ will not entirely be related to the training itself. Students will talk to each other in the class, and, especially if one of the informal leaders (‘opinion maker’) in this class stops smoking, this will affect other students and they will be more likely to stop smoking as well. From a public health perspective, this is a most welcome effect. Technically speaking, however, this is an undesired effect, because it will blur the effects of the intervention; students in the control group will show an effect as well. In other words, individual randomization for the purpose of testing behavioral interventions in larger groups will likely result in an underestimation of the treatment effect. An alternative is the use of cluster randomization [17]. Here, clusters of individuals are randomized rather than individuals themselves, for example wards or clinics.

In our cluster randomized study, we randomized wards. Two parallel groups are compared and the intervention is expected to be superior to the control arm. The setting of this study is an academic hospital: University Medical Center Leipzig in Leipzig, Germany. Informed consent is obtained from each study participant after admittance to the ward, before the baseline assessment.

### Intervention arm (arm one)

The intervention is a stepped care model to provide targeted psychosocial help to cancer patients and consists of three steps.

Step one: each patient is screened for distress. The results of this screening are electronically computed, graphically visualized, and fed back to the clinician in charge. Patients’ level of distress is visualized by colors analogue to traffic lights: green = no or little distress, yellow = medium level of distress and red = high level of distress). The doctor can also view the detailed numeric results.

Step two: if the patient is at a medium or high level of distress, the doctor performs a brief interview along with their routine consultation with this patient. Together, they should discuss the psychosocial problems the patient currently has and decide on further steps. This second step is a key element in the stepped care model. It ensures the following: a) that the patients have access to the doctor to discuss their emotional problems, which is an important supportive care need for many patients [18], b) ‘false positives’ can be detected early, c) the clinician remains the key person for decisions on patient care and d) the patients can express their needs or concerns regarding psychosocial support. Studies show that this procedure is well accepted by patients and physicians [19, 20]. As doctors frequently feel insufficiently prepared to talk with patients about their emotional problems, and sometimes avoid such consultations, they are trained individually to perform this task. They learn how to, within the limited consultation time, ask questions, respond to patient concerns, consider their supportive care needs and provide further help.

Step three: if the patient and doctor decide that psychological support is needed, the hospital’s psycho-oncological consultation liaison (CL) service is informed and provides mental healthcare. If the patient has financial, vocational or other social problems, the hospital’s social service is called. If necessary, further support in the outpatient setting when the patient is discharged from the hospital is organized by these two teams.

Quality assurance: the team of this CL service consists of psychologists with formal training in psychotherapy, either cognitive-behavioral or psychodynamic. The social services team consists of professionally trained social workers. Both CL and social services provide support according to the current guidelines. The psychotherapists receive regular supervision by a fully trained senior psychotherapist. Adherence to the stepped care model is monitored by asking the patients whether their doctor has discussed emotionally relevant topics.

### Care as usual arm (arm two)

Patients who are treated on wards in the control arm receive care as usual. This means, doctors can call CL service and the hospital’s social service whenever they feel it is necessary. Doctors do not receive specific training to detect distress or to talk with the patients about emotional problems.

### Data collection

The primary endpoint mental health is ascertained with the Hospital Anxiety and Depression Scale (HADS) [21]. HADS identifies depressed patients with a sensitivity of 0.96 [22]. Social problems are measured with the European Organisation for Research and Treatment of Cancer Quality of Life Questionnaire Core Instrument (EORTC QLQ-C30), using the social functioning and role functioning scales [23]. Satisfaction with care is measured with the Quality of Care from the Patients Perspective questionnaire (QPP) [24], psychiatric comorbidity is measured with the Structured Clinical Interview (SCID) [25]), and the utilization of medical care is measured with the questionnaire used in the German Federal Health Survey [26]. Clinical data are obtained from the medical records. The provision of CL services and/or social services is ascertained from the hospital information system.

The patients are interviewed at the beginning (t1) and the end (t2) of the hospital stay, three months after baseline (t3) and six months after baseline (t4). At t1 and t2, data collection is done electronically with the help of tablet computers. At t3 and t4, it will be done via phone or face-to-face, depending on the patient’s preference. For patients who prefer paper-based data collection, this is possible as well. The equivalence of both methods is well documented [27, 28, 16]. The Computer-Based Health Evaluation System (CHES) software (Evaluation Software Development, Innsbruck, Austria) is employed for electronic data capture [29]. Electronic data capture ensures that no missing data are in the data set unless the patient quits the study. Study nurses with an academic psychology education contact the patients, inform them about the study, obtain written informed consent and perform data collection. Patients in the control wards undergo the same assessments as patients in the experimental wards.

### Eligibility

Patients aged 18 years or older of both sexes and with all types and stages of cancer are eligible for this study. They must be admitted to the University Medical Center Leipzig for diagnosis or treatment of cancer in one of the following departments: Urology, Pneumology, Maxillofacial Surgery, Radiation Oncology, Gynecology, Neurosurgery, Visceral Surgery, Orthopedics, Gastroenterology, or Laryngo-Rhino-Otology. Patient exclusion criteria are insufficient command of German and no written informed consent.

### Sample size

At the University Medical Center Leipzig, 2,000 patients with malignant diseases are treated per year according to the local cancer registry. Of those, about 350 are ineligible for the study because of age or because they are treated at a ward that is not participating in this study, leaving 1,650 eligible patients per year and 137 per month. Thus, about 1,200 patients can be approached initially. Assuming that 20% of patients will decline participation at t1, this leaves about 1,000 patients participating at baseline. Based on previous experience from a large epidemiological study with cancer patients at the University Medical Center Leipzig [22], we assumed that 20% of the patients would drop out during the follow-up period due to death or withdrawal from the study, leaving 800 patients at t4.

Cluster randomized trials must take into account the within- and between-cluster variation by increasing the sample size [30]. The between-cluster-variation coefficient could only be approximately estimated because of a lack of data in the literature. We therefore used the recommendation of Hayes and Bennett [30] and assumed k = 0.2. We further assumed an equal cluster size. In our trial, 13 clusters are included and randomized. The low number of clusters was considered in the sample size calculation with a specific formula (t-distribution). Data from a previous large study in the same hospital [22] suggests that the average HADS score in cancer patients is 13, with a standard deviation of 7. This is the expected mean HADS score in the control arm at t4. With 13 clusters, k = 0.2, α = 0.05, a power of 80%, and n = 400 per arm, a delta in HADS scores of 5.5 can be identified. If k should be k = 0.1, a difference of 4.1 can be detected.

### Measures to reduce confounding and bias

Potential confounding will be controlled by randomization. Randomization was completed externally by the Center for Clinical Trials at University Medical Center Leipzig, Germany. In order to reduce baseline differences, the randomization was stratified according to the average frequency of psycho-oncological CL services in the past two years per department. This information was taken from the yearly documentation of the CL service.

By using standardized tools and training interviewers, a potential information bias will be reduced. Moreover, the study nurses collect data either always on intervention wards or always on control wards; they do not change trial arms. The patients are not told to which group they have been randomized. However, the intervention itself (screening, consultation with the doctor and referral to the CL service) obviously cannot be blinded. The results of the randomization can also not be concealed to the doctors because they have to change their consultation behavior in the intervention arm.

Selection bias is intended to be kept to a minimum through high participation rates. Based on previous experience [31], we know that highly distressed patients decline participation disproportionately more often. We control this effect by documenting the reasons for decline and then comparing participants and non-participants.

### Statistical analysis

The main analysis will compare HADS scores of all patients at t4 in arm one versus arm two using t-tests. Subsequently, only patients highly distressed at baseline in the two groups will be compared regarding their HADS score at t4. Because of the limited number of departments, complete control for confounding cannot be guaranteed despite randomization; therefore the following variables will be subsequently adjusted for in a mixed-model analysis: sex, age, stage of disease and baseline distress. The analyses will be done as according to the intention to treat principle. No interim analyses are planned.

### Ethics, data protection and study registration

Ethical approval for this study was obtained from the Ethics Committee at Leipzig University (reference number 210-12-02072012). As this is a cluster randomized trial, patients cannot be asked whether they agree to be randomized or not. Instead, informed consent is obtained after the patient has been admitted to the ward. Patients can opt out, that is, they can decide not to take part in this study. If this is the case, the patient is not screened for distress and, hence, the following steps of the stepped care model are not applied. These patients receive care as usual.

Confidentiality of the data is ensured by using pseudonyms (patient identification numbers) with each questionnaire and data form. No person-identifying information will be stored together with the medical and patient-reported outcome data. Patient identification numbers and person-identifying information (address and telephone number) are stored separately in a locker, physically unlinked to the other data. Only the principal investigator and the two researchers employed in the project have access to the data. The study has been registered with the Clinical Trials Register clinicaltrials.gov (identifier: NCT01859429).

### Monitoring

The principal investigator (SS) receives monthly written and oral reports about enrolment and study conduct. The project manager (HD) monitors data collection on a daily bases and supervises all study nurses. He trained the doctors and visits them regularly on the wards to ensure that they adhere to the study protocol. They in turn can address him with any request they might have at any time. The study nurses are onsite daily at the wards and, in arm one, ensure that the doctors make use of the screening results.

### Dissemination policy

Trial results will be communicated to the study participants after data collection has been completed. Each individual will receive a letter with information about the study results in a way that is easily understandable for lay persons. Healthcare professionals and investigators will be informed via a scientific paper and presentations at conferences. All collaborators who have participated in the trial design and who enrolled patients are eligible for authorship. A press release will inform the public.

## Discussion

Mental health problems not only cause emotional suffering but also direct and indirect costs related to financial, social and clinical issues [32, 33]. They are related to high utilization of health services and can even result in increased mortality [1]. This effect is probably due to poor therapy adherence. A meta-analyses by DiMatteo *et al*. [2] showed that patients suffering from clinical depression tend to fail in their oncological treatment three times more often than others.

This calls for timely and adequate psychosocial support, especially as we know that such support is effective [34]. However, not every cancer patient can and must be treated by a mental health professional. Allocating limited resources most sensibly and economically is of crucial importance for our healthcare system to ensure the best quality of care to as many patients as possible. It is the hope of STEPPED CARE, that this model is both effective and efficient and that it can be tested and implemented in other hospitals as well, if proven to be effective.

## Trial status

The trial is ongoing. Patient enrolment has started (October 2012) and is not yet complete. It is expected to end in December 2014.

## Authors’ information

SS: Psychologist and epidemiologist; Chair of the German Society of Psycho-Oncology. HD: Psychologist. SB: Gynecologist; Head of the Breast Cancer Center at Leipzig University. ArD: General surgeon. AnD: ENT surgeon; Chair of the German Society of Head and Neck Oncology. JE: Gynecologist. KP: Radiation oncologist. FL: Medical Oncologist; Head of the University Cancer Center of Leipzig University Hospital. JüM: Neurosurgeon. JoM: Gastroenterologist. DN: Medical Oncologist. TP: Orthopedist. FS: Maxillofacial surgeon. J-US: Urologist. HW: Pneumologist. AK: Psychotherapist.

## Abbreviations

CL: consultation liaison
EORTC: European Organisation for Research and Treatment of Cancer
HADS: Hospital Anxiety and Depression Scale
SCID: Structured Clinical Interview
t: time point.
